# Determinants of nutrition knowledge, attitude and practices of adolescent sports trainee: A cross-sectional study in Bangladesh

**DOI:** 10.1016/j.heliyon.2021.e06637

**Published:** 2021-04-06

**Authors:** Md. Bakhtiar, Md. Masud-ur-Rahman, Md. Kamruzzaman, Nargis Sultana, Shaikh Shahinur Rahman

**Affiliations:** a Department of Exercise Physiology, BKSP, Zirani, Savar, Dhaka, Bangladesh; b Protibondhi Sheba o Sahajjo Kendro Magura, Ministry of Social Welfare, Bangladesh; c 250 Bedded General Hospital, Meherpur, Bangladesh; d Department of Applied Nutrition and Food Technology, Islamic University, Kushtia, 7003, Bangladesh

**Keywords:** Nutrition, Knowledge, Attitude, Practices, Athletes, Sports

## Abstract

**Background:**

Nutrition is an important predictor of an athlete's performance. It is more important for particularly those athletes who are in the growing stage (adolescents). This study aimed to assess their current level of knowledge, attitude, and practices (KAP) of nutrition and to predict potential factors impacting their level of these KAP.

**Methods:**

A cross-sectional study was conducted among adolescent trainee athletes selected conveniently from 11 sports departments (e.g. cricket, football, hockey, etc.) of Bangladesh Krira Shikkha Protishtan (BKSP). A semi-structured interviewer-administered questionnaire was used for data collection. Anthropometric measurements (e.g. height, weight, BMI, BF %) were taken using appropriate methods. The data were analyzed using R (v3.6.1) and Python Jupyter Notebook. Descriptive statistics, t-test, analysis of variance (ANOVA), and logistic regression were used to determine the relationship between dependent and independent variables.

**Results:**

A total number of 260 participants were enrolled in this study, most of them (86%) were male. Their mean age was 15.50 ± 1.83 ranging from 12-19 years. More than half of the participants had good nutrition knowledge (n = 149, 57.3%). Fifty-seven percent of participants had a positive attitude (n = 146) and 57.69% (n = 150) had a good level of practices regarding nutrition. Age (p = 0.007), gender (p = 0.004), department of training (p = 0.0034), and duration of sports training (p = 0.004) of participants were significantly associated with knowledge, while only age and BMI were significantly associated with practices. Athletes with less body fat were more likely to have good nutrition practice behavior (AOR 0.895; 95% CI 0.83, 0.96; p < 0.05) and nutrition knowledge was positively associated with nutrition practice (AOR 2.335; 95% CI 1.405, 3.88; p = 0.001).

**Conclusion:**

Knowledge, attitude, and practices level observed among many of our participants was satisfactory. Previous nutrition training was found as a potentially modifiable factor of good nutrition knowledge and good nutrition knowledge was found to be a predictor of good practice score. Thus, it is necessary to provide appropriate nutrition information to adolescent athletes through proper educational training and intervention on a regular basis.

## 1 Introduction

Nutritional status is one of the determinants of physical fitness and training of a sports person [1, 2]. Proper nutrient intake improves physical performance, on the other hand, nutrient deficiency consequences poor athletic performance [3, 4]. Healthy dietary patterns and adequate nutrition are necessary for an adolescent's life, especially those who are involved in sports [5]. Previously, only elite athletes were concerned regarding proper nutrition for their performance [6], however, concern about the importance of nutrition as an integral part of the training has been increased among most athletes [4, 7]. In the adolescent stage, energy requirement increases and varies based on gender and activity [8]; those higher demands are poorly met in adolescent athletes [5, 8, 9].

Students at BKSP receive a general education (from school to university degree level) along with specialized sports training. The institute is fully residential and students are served food, medical treatment, and sports materials [10]. Energy requirements of adolescent sports trainees are generally high due to their increased physical activity, and body growth [11]. Therefore, when nutrient intake is inadequate, it leads to poor athletic performance, and additional health problems [11]. To avoid illness, injury and to fuel properly in each step (before, during and after) of a sporting event, education on sports nutrition is necessary [8]. Additionally, variation in culture, race, religion, economic status, and type of sport leads to variation in nutritional practices [11]. However, several environmental factors (busy schedule, travel and food culture) also hinder athletes from getting the required dietary practices. Nutrition knowledge also influences attitude and eating behavior [12]. Adolescent sports trainee often relies on their coaches for nutrition guidance despite gaining brief information from their regular textbooks. Dependence on the internet search for proper diet and nutrition is growing, however, it's difficult to separate authentic information. Lack of proper training on diet and nutrition creates potential harm if the coaches and athletes are misinformed [13]. This demonstrates the importance of proper nutrition knowledge and healthy dietary practices for adolescent athletes as well as for the coaches. Therefore, adolescent athletes and their trainers are the appropriate target group for nutrition education as they have the potential for positive changes [14].

An adolescent from low-income communities lacks educational materials which result in insufficient knowledge of sports nutrition and supplementation to make health-conscious decisions. The effect of education on nutrition knowledge and attitude has also not been studied well. Though the study suggests that short-term education on nutrition can enhance supplementation of knowledge [15]. Therefore, the study was designed to find out gaps and areas of knowledge deficits before an intervention to put in place. This study aims to assess nutrition knowledge, attitude, and practices of young adolescent athletes and their interrelation. Moreover, potential determinants of knowledge, attitude, and practice will be explored.

## 2 Methods

### 2.1 Study design and subjects

**Figure 1:**
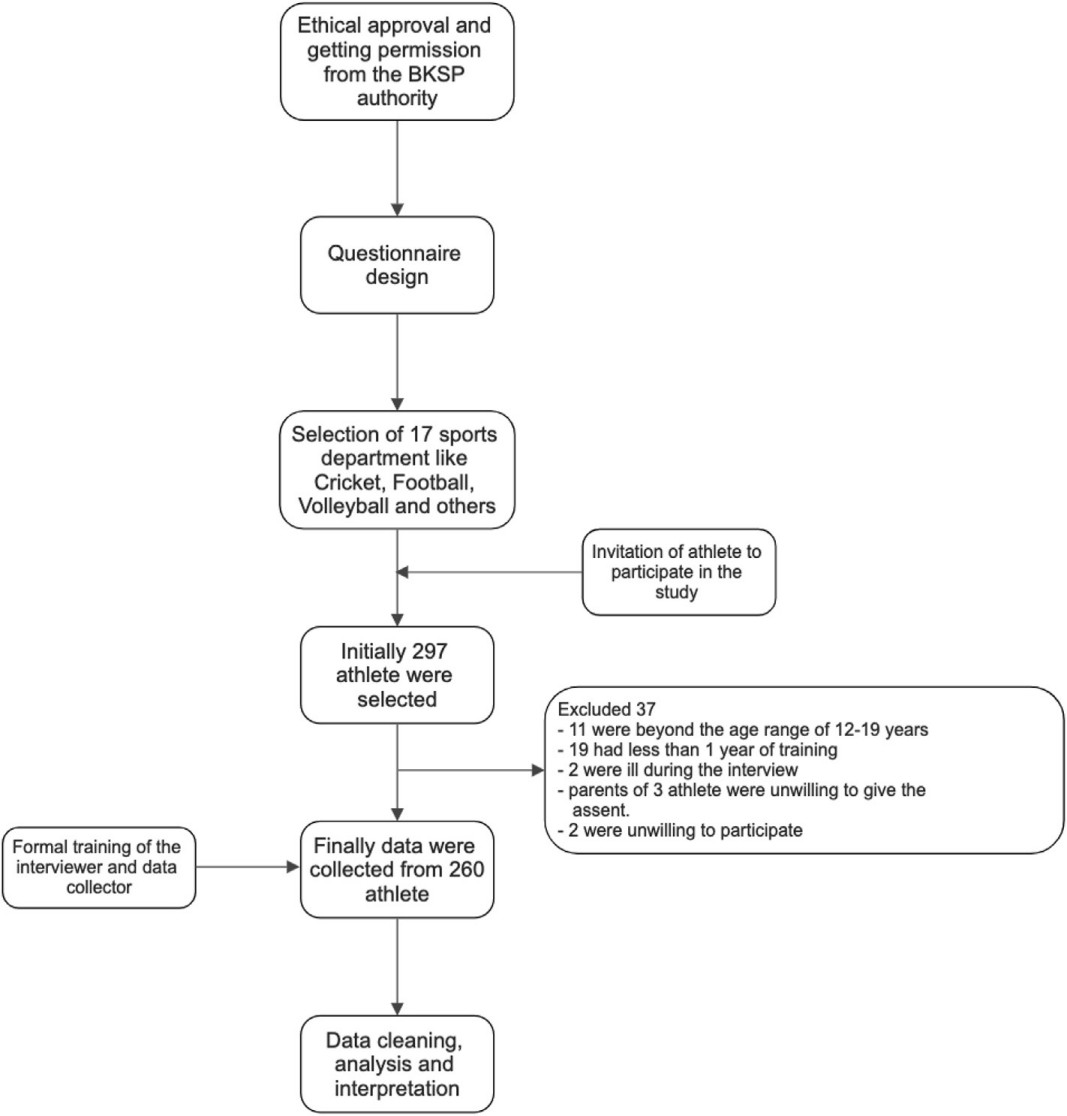
Flowchart of study design.

The current study was a cross-sectional study conducted at BKSP (Bangladesh Krira Shikkha Protisthan) among adolescent trainee athletes from the different disciplines of sports. The study was conducted from September 2017 to March 2018 and adolescent trainee athletes of different sports departments such as cricket, football, volleyball, and others of BKSP were included. First, different sports departments were chosen then samples from each of the sports departments were chosen conveniently, inviting all of the adolescent trainee, during the visit after the end of their regular training session. Adolescent trainees athletes participating in different games, aged between 12 to 19 years, were included in this study. As one of the aims of this study was to investigate the impact of training on their KAP, participants who had less than one year of training in BKSP were excluded. Participants who were critically ill were excluded from this study. Participants who were reluctant to take part were also excluded (Figure 1).

### 2.2 Compliance with ethical standards

This research was conducted with the ethical approval [reference no: FBS/ERC/2018] of the ethical review committee of the Faculty of Biological Science of Islamic University, Kushtia, Bangladesh. The study was conducted in accordance with the 1964 Declaration of Helsinki and its later amendments. The aim, potential risk, benefits, and confidentiality were made known to respondents in an informed consent form. Additionally, participation was voluntary and we gave them full right to refuse or accept to participate. Anonymity and confidentiality of the respondents' data were assured. Informed written consent for participants under 16 years old was taken from the respected coaches of each sports department and parent's permission was taken contacting them over the phone.

### 2.3 Survey questionnaire design

Prior to this study, a questionnaire targeting information on nutritional knowledge, attitude, and practice was developed by bringing together a searched summary of peer-reviewed published literature and then panel discussion with an expert group. The developed structured interviewer-administered questionnaire was further gone through pretesting and necessary modification. The questionnaire consisted of several parts, including demographic information of participants, details of anthropometric measurement, and information on knowledge, attitude, and practices towards nutrition. The first section was designed to get information about the demographic characteristics of students and any prior exposure to nutrition training and the sources from where the athletes did get those training and/or information on nutrition. Anthropometric data such as participant's height, weight, BMI, and body fat percentage (%) were also collected under this section. The second, third, and fourth sections include the information on knowledge regarding nutrition, attitude towards nutrition and practice respectively. The questionnaires for the knowledge, attitude and practice section were adapted from a previous study with modification to consider the context of regional difference [15, 16, 17, 18, 19]*.* The questionnaires for knowledge, attitude, and practices were then discussed within our research group, and tailored to the local context, and translated into the Bengali language. To validate, the questionnaire was first pretested in BKSP. A total of 20 athletes was included in the pretest and the number of participants was around 7.5 % of the total number of participants included in the final study. Based on the pretesting results, face validity was established by consulting experts from home university and BKSP. Required changes were made in the questionnaire to transform it friendly to the respondents and minimize the respondent burden. The question set in all of the three parts, knowledge, attitude, and practice, were close-ended (yes or no and on the Likert scale), which leave the chance of confirmation bias to a minimum. Moreover, after the pretest, discussion with several experts helped minimize confirmation bias. Finally, the questionnaire was checked further to ensure internal consistency (Cronbach's alpha = 0.83).

### 2.4 Knowledge, attitude, and practices (KAP) scoring

To represent the results of knowledge, attitude, and practices (KAP) status towards nutrition, based on the response received from the participants (sample questions are provided as supplementary file), a detail scoring system was established. The knowledge section had 22 statements which could be answered as “true” or “false”. Each correct response scored as “1,” and the incorrect one as “0”. In the attitude section, 11 statements were given, which could be answered on a Likert scale ranging from “strongly agree” to “strongly disagree”. The scores ranged from “1–5”, where “5” was the most positive and “1” was the most negative. In the practice section, 15 statements were prepared with their response as “yes” or “no”. Each positive response in this section scored as “1” and negative responses were scored as”0”. After scoring, the score for all of the answered questions for Knowledge, Attitude, and Practice was summed up separately. This score means that the more the score the more the participant has good knowledge score and practice habit and have a positive attitude. Overall, a positive attitude means being confident about the effect of nutrition and food on sports performance. People with positive attitudes can remain aspirant and more aware of their nutritional intake or nutrition of the food they consumed [20]. To decide the individual as having a good or bad knowledge score and practice habit and a positive or negative attitude towards nutrition, the median score was used as the cutoff point. Though there is criticism, the median split can create a group with the best result when the variable is continuous and normally distributed [21].

### 2.5 Interview and data collection

Before the data collection, the Principal Investigator (PI) visited the study area particularly all sports departments, and arrange a short meeting with corresponding coaches. The researchers and postgraduate diploma students at BKSP (as data collectors and enumerators) were involved in the data collection procedures. Data collectors were given a day-long training to familiarize them with the objective and relevance of the study, confidentiality of information, participants’ rights, and informed written consent. They were also given proper training on how to minimize social desirability bias [22] and to handle flat slope syndrome [23]. Three graduate colleagues from BKSP supervised the data collection procedures. One session was taken for athlete, and each session to take an interview and anthropometric measurement of each athlete took around 40–60 min.

### 2.6 Anthropometric assessment and body composition analysis

The same instructions were read to each participant to ensure that the purpose of the study was clearly understood by the participants. Attached to each questionnaire were a cover letter and consent form to be signed by the participant or their corresponding coaches. All of the data collectors and enumerators were trained well for anthropometric measurement to minimize measurement error. Height and weight were measured according to standard procedures [24] described elsewhere using a stadiometer (Charder's HM230 ′Wall Mounted Manual Stadiometer, USA) and a standard weighing scale (Camry – EB9063, China). Height was measured to the nearest 0.2 cm without wearing the shoes. Weight was measured to the nearest 0.1 kg using a calibrated electronic scale (Tanita, Tokyo, Japan) with minimum clothing. BMI was measured from weight and height using a standard formula. Skinfold calipers (Harpenden Skinfold Caliper, Model: HSK-BI, USA) was used to measure skinfold thickness on five sites (triceps, bicep, subscapularis, abdominal, and calf) of the body. Then from skinfold thickness, body fat percentage was calculated using the standard formula proposed by Wagner Luis Ripka *et al.* (2017) [25].

### 2.7 Statistical analysis

Data were first entered in the excel sheet and finally analyzed using RStudio (R version 3.6.1) and Python Jupyter Notebook. Correlation between Knowledge, Attitude, and Practice score was compared using Pearson's correlation coefficient. The normality of the data was tested using Q-Q-plot and Shapiro-Wilk test and Levene test was used to test homogeneity of variance. Means KAP score based on related factors were compared using one-way analysis of variance (ANOVA). Post hoc tests (Tukey) were used for determining the mean differences within the groups. Kruskal-Wallis rank-sum test, a nonparametric alternative of one-way ANOVA, was used when the assumption of equal variances and assumption of normality was violated. Nutrition knowledge, attitude, and practices between dichotomous variables were compared using an independent sample t-test and Wilcoxon rank-sum test was used when assumptions were violated. Moreover, factors/variables that lead to good or poor KAP scores were analyzed using binary logistic regression. Multivariable logistic regression was used to examine differences in nutrition knowledge, attitude, and practice behaviors (good vs poor and positive vs negative) based on age, gender, weight, height, BMI, percent body fat, and dept. of training). A backward stepwise regression procedure was used to develop the perfect model containing variables that are statistically significant. Associations are quantified by odds ratios (OR) with 95% confidence intervals (CI) and results were adjusted for all of the variables included in the model. A p-value of ≤0.05 was used as significant for statistical results.

## 3 Results

A total number of 260 adolescent trainee athletes from different sports department of BKSP participated in this research. All the participants were below 19 years of age. The mean ± SD (Standard Deviation) age of our participant was 15.50 ± 1.83 with a range of 12–19 years. Most of the participants were male (n = 233, 89.62%), while few were female (n = 27, 10.38%). Moreover, according to the religious category, Muslim (n = 233, 89.62%) were predominant followed by Hindu (n = 21, 8.08%), Christians (n-5, 1.92%) and Buddhists (n = 1, 0.38%). Most of the participant were school-going (secondary school) adolescents (n = 161, 61.9%) followed by the college (higher secondary) (n = 98, 37.7%) and degree or university students (n = 1, 0.4%) (Table 1).

**Table 1:** Demographic characteristics of the survey respondents (n = 260).

Characteristics	Frequency	Percentage
**Age** (15.50 ± 1.83) years ≤15 >15	131 129	50.4 49.6
**Gender** Male Female	233 27	89.62 10.38
**Religion** Islam Hindu Buddhist Christian	233 21 5 1	89.62 8.08 1.92 0.38
**BMI Classification (Cut-off)** Underweight (<18.50) Normal (18.50–24.99) Overweight (≥25) Obese (≥30)	53 204 3 0	30.4 78.5 1.2 0
**Educational Level** (Current) High school (Secondary) College (Higher Secondary) Degree/University (Bachelors)	161 98 1	61.9 37.7 0.4
**Department of Training**		
Cricket	42	16.2
Football	36	13.8
Athletics	37	14.2
Basketball	25	9.6
Volleyball	20	7.7
Archery	27	10.4
Table Tennis	21	8.1
Karate	10	3.8
Wushu	14	5.4
Taekwondo	8	3.1
Hockey	20	7.7

BKSP has 17 distinct departments where athletes were being trained according to their capacity and guidance from the coach. In the current research, data were collected from 11 departments following the inclusion criteria using a convenient sampling technique. Most of our participants were from the department of cricket (n = 42, 16.2%) followed by athletic (n = 37, 14.2%), football (n = 36, 13.8%), archery (n = 27, 10.4%) and so forth. Due to the limitation of time, we could not reach all the departments. The number of participants, according to the departments of sports, are shown in Table 1.

The mean ± SD (standard deviation) of the training duration was 3.67 ± 2.29 years ranging from 1 to 13. Most of our participants were young trainee athletes (Table 3). The majority of our participants had their training period in their corresponding sports department equal to 5 years or less (n = 206, 79.2%). Nearly 21 percent of respondents received training for more than five years.

### 3.1 Anthropometric, and body composition details

The mean ± SD weight of our respondents was 57.31 ± 9.41 kg ranging from 25 to 83 kg. The mean ± SD (Standard Deviation) height of the participants was 5.47 ± 0.38 feet ranging from 4.10 to 6.50 feet. In this study, the mean ± SD (standard deviation) BMI was 20.07 ± 2.05 ranging from 13.80 to 25.70. Most of our participants belong to the healthy category of BMI (n = 204, 78.5%). Around one-third of the participant were underweight (n = 53, 30.4%), however, only 3 (1.2%) were overweight and no obese individuals (Table 1). The mean ± SD (Standard Deviation) body fat percentage (%) of all respondents was 9.06 ± 4.59 ranging from 4.10 to 25 (Table 3).

### 3.2 Nutrition education

Participants were asked if they had any previous training or attended in seminar/workshop on nutrition. A vast majority of participants replied that they never had any nutritional education or training during their sports training period (n = 233, 89.6%). Participants, who had prior nutrition training, were further asked how long ago they had their last training or education in nutrition. Only 27 participants (10.4%) had previous exposure to nutrition training; among them, only ten participants had their training three months ago. Six of them have had their previous nutrition training one year before (Table 2).

**Table 2:** Nutrition education among survey respondents (n = 260).

	Frequency	Percentage
**Number of nutrition training** Once Twice Thrice ≥4^th^ time Never	15 6 6 0 233	5.8 2.3 2.3 0 89.6
**Duration of Training (Years)** ≤5 >5	206 54	79.2 20.8
**Time since last training was received (n = 27)** 1 month ago 3 months ago 6 months ago 1 year ago	3 10 8 6	1.2 3.8 3.1 2.3
**Source of Information** Book Magazine Newspaper Teachers Coach Trainer Doctor Nutritionist Parents Teammates Internet Others No source	97 5 9 27 78 3 7 13 10 4 6 1 0	37.3 1.9 3.5 10.4 30.0 1.2 2.7 5.0 3.8 1.5 2.3 0.4 0

### 3.3 Source of information on nutrition

Nearly forty percent of respondents got information about nutrition from the textbooks (n = 97, 37.3%). Thirty percent replied that coaches were the primary source of information (n = 78), followed by teachers (n = 27, 10.4%). Very few of them mentioned other reliable sources such as doctors, nutritionists, trainers, health magazines, and the internet. Details are shown in Table 2.

### 3.4 Nutrition knowledge

**Figure 2:**
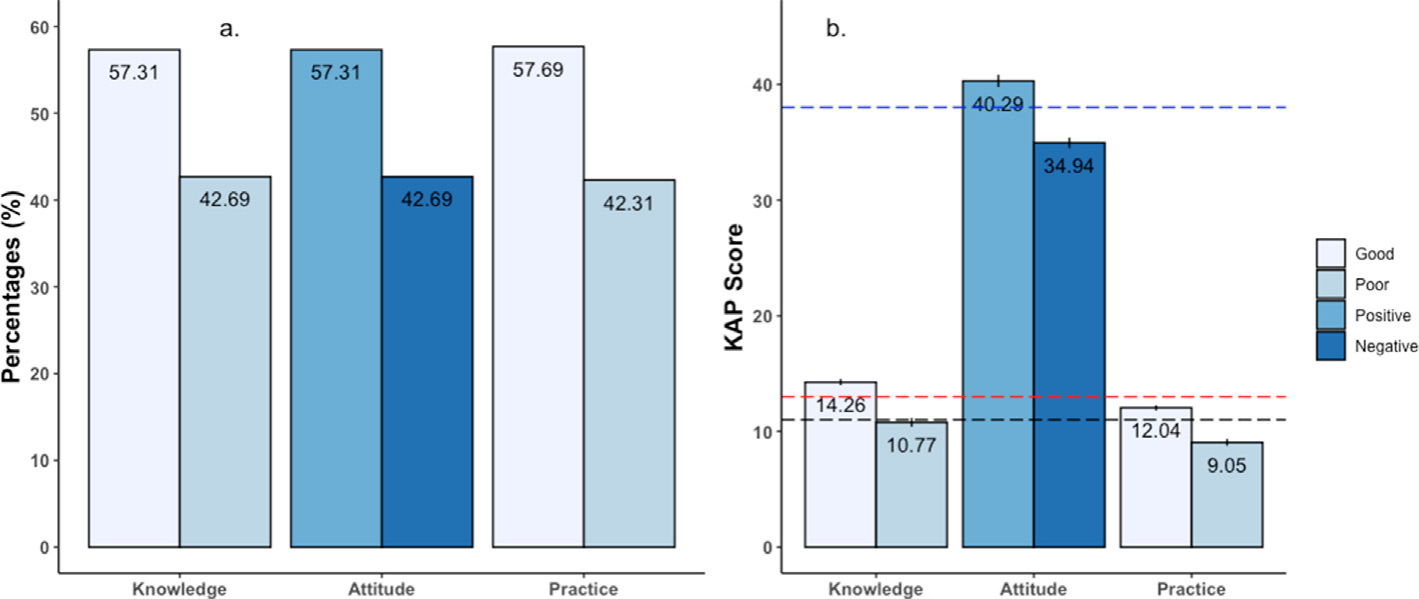
Overall Nutrition Knowledge, Attitude and Practices (KAP) Score with percentages of participant with binary category of KAP score (Picture a.) and Mean KAP score of participants (Picture b.). (∗Each bar represents mean and 95% CI of mean and the red, blue and black horizontal line represent median score of knowledge (13), attitude (38) and practice (11)).

The mean ± Standard Deviation (SD) and median score of nutrition knowledge for the entire sample were 12.77 ± 2.17 and 13 respectively (Figure 2, Table 3). The highest score was 18, while the lowest score was 5. More than half of the participants had good nutrition knowledge (n = 149, 57.3%) whereas nearly forty-three percent of respondents had a poor knowledge level on nutrition (n = 111, 42.7%) (Figure 2).

**Table 3:** Anthropometric, body composition and nutrition education characteristics of athletes.

	Mean ± SD	Median (Range)	Normal distribution
Age (Years)	15.50 ± 1.83	15 (12–19)	No
Weight (kg)	57.31 ± 9.41	58 (25–83)	Yes
Height (cm)	66.51 ± 4.23	66 (50–82)	No
BMI (kg/m^2^)	20.01 ± 2.32	20.01 (13.29–26.66)	Yes
Body Fat (%)	9.01 ± 4.60	8.1 (4.1–25.0)	No
Training duration (Years)	3.67 ± 2.29	3 (1–13)	No
Knowledge score	12.77 ± 2.17	13 (5–18)	Yes
Attitude score	38.00 ± 3.32	38 (28–46)	Yes
Practice score	10.77 ± 1.82	11 (6–15)	No

Age had a significant relationship with nutrition knowledge score (Table 4). Participants, more than 15 years of age, had a better level of knowledge than their juniors (p = 0.007). Their mean ± SD knowledge score was 13.13 ± 2.13, which was higher than the other age group (<15 years) (Table 4). The educational level of the participants did not show any significant influence on their nutritional knowledge. Participants who were studying in schools has a similar level of knowledge score with those who were studying in college (p = 0.619). There was also a significant relationship between knowledge score, and the department of training (p = 0.003). Cricket, football, and wushu trainee adolescents had a relatively higher level of knowledge than the other group of participants.

**Table 4:** Bivariate analysis of nutrition knowledge, attitude and practices (n = 260).

Variables	Knowledge		Attitude		Practices	
	mean ± SD	P value	mean ± SD	P value	mean ± SD	P value
**Age group** ≤15 >15	12.40 **±** 2.16 13.13 ± 2.13	**0.007∗**	37.81 **±** 3.18 38.19 ± 3.45	0.361	11.11 ± 1.81 10.43 ± 1.77	**0.003∗**
**Gender** Male Female	12.92 ± 2.12 11.81 ± 2.35	**0.004∗**	38.11 ± 3.35 37.33 ± 3.08	0.193	10.82 ± 1.85 10.41 ± 1.61	0.206
**Educational Level** High school (Secondary) College (Higher Secondary) Degree/University ^**#**^	12.71 ± 2.24 12.87 ± 2.08 11.00 ± 0.00	0.619	37.81 ± 3.36 38.31 ± 3.25 38.00 ± 0.00	0.500	10.86 ± 1.88 10.61 ± 1.71 11.00 ± 0.00	0.541
**Department of Training** Archery Athletics Basketball Cricket Football Hockey Karate Table Tennis Taekwondo Volleyball Wushu	11.9 ± 1.80 12.4 ± 2.67 12.0 ± 1.86 13.5 ± 1.98 13.0 ± 1.80 12.9 ± 1.83 12.6 ± 2.32 12.8 ± 2.14 12.9 ± 1.46 12.2 ± 2.80 14.6 ± 1.70	**0.0034**	38.2 ± 3.47 38.5 ± 3.54 37.8 ± 2.68 37.5 ± 3.07 37.8 ± 3.62 38.1 ± 4.46 37.3 ± 3.27 38.0 ± 3.02 37.4 ± 2.00 38.9 ± 1.86 38.3 ± 4.61	0.929	10.7 ± 1.54 10.7 ± 1.81 11.0 ± 2.17 10.6 ± 1.87 11.1 ± 1.81 11.0 ± 1.17 9.6 ± 1.35 11.2 ± 1.81 10.8 ± 1.91 11.0 ± 2.00 9.79 ± 2.04	0.256
**Body Fat (%) (9.055 ± 4.59)** Male (8.99 ± 4.54) Better (7.62 ± 2.58) Good (16.78 ± 1.95) Overweight (22.39 ± 1.49) Female (9.53 ± 5.16) Acceptable (23.4 ± --) Better (8.041 ± 2.96) Good (20.5 ± 2.12)	12.97 ± 2.09 11.84 ± 2.95 12.8 ± 1.93 13 ± --11.83 ± 1.99 11.5 ± 0.707	0.4352 0.2419	38.21 ± 3.29 36.31 ± 2.16 39.8 ± 3.88 40 ± --36.95 ± 3.55 35.5 ± 3.53	0.060 0.568	10.91 ± 1.81 10.36 ± 1.42 10 ± 1.49 10 ± --10.42 ± 2.18 8.5 ± 0.71	0.059 0.3706
**BMI** Underweight Normal Overweight	12.81 ± 1.95 12.72 ± 2.22 15.33 ± 2.08	0.116	37.54 ± 3.22 38.12 ± 3.34 38.00 ± 3.60	0.534	11.50 ± 1.50 10.58 ± 1.85 10.33 ± 1.33	**0.004∗**
**Duration of Training** ≤5 years >5 years	12.63 ± 2.22 13.29 ± 1.91	**0.004∗**	38.30 ± 3.14 37.62 ± 3.24	0.353	10.84 ± 1.85 10.48 ± 1.69	0.186
**Nutrition Training** Once Twice Thrice or more Never	13.26 ± 2.08 13.33 ± 1.05 13.67 ± 6.07 12.69 ± 2.19	0.481	39.26 ± 3.95 38.16 ± 1.72 39.83 ± 2.71 37.87 ± 3.30	0.481	10.26 ± 1.44 11.33 ± 2.06 10.66 ± 1.63 10.79 ± 1.84	0.620

∗ = Statistically significant with value ≤0.05 and highlighted bold, #There were only one athlete completed university

Nutrition scores among participants with different BMI groups were not significantly different (p = 0.116) (Table 4). Moreover, the difference in nutrition knowledge score among participants with different levels of body fat was not significant and this insignificant difference was true for both male and female athletes (Table 4) (0.4352 and p = 0.2419). Participants with more than five years of training had a higher level of knowledge in nutrition than the participant who had less than or equal to 5 years of training (p = 0.044). Whereas knowledge score among participants with a different frequency of nutrition training was not significantly different (p = 0.481) (Table 4).

### 3.5 Attitude towards nutrition

The mean ± SD and median score for attitude towards nutrition for the entire sample were 38.00 ± 3.32 and 38 respectively. The highest score was 46, whereas the lowest was 28 (Table 3). More than half of our participants had a positive attitude towards nutrition (n = 149, 57.31%), while the rest had a negative attitude (n = 111, 42.69%) (Figure 2).

Unlike knowledge score, attitude score was not significantly different among participants of different age groups (p = 0.361, Table 4), though participants more than 15 years of age had a better level of attitude score than their juniors. Moreover, boys had a better attitude towards nutrition than girls, but the finding was not statistically significant (p = 0.193). The educational level of the participants did not have any influence on their attitude towards nutrition. Participant who was studying in college had a higher level of mean attitude score than those who were studying in schools, but the relationship was not statistically significant (p = 0.500). Athletes of different department of training didn't show a significantly different attitude score (p = 0.929). BMI of our participant also did not show any significant relationship with their attitude towards nutrition (p = 0.534). The participant with different body fat (BF %) didn't show to have a significantly different attitude score, and this is true for both male and female athletes (p = 0.060 and p = 0.059). Moreover, participants with different duration of training and participants with a different frequency of training shown to have a similar attitude score (p = 0.353, p = 0.481) (Table 4).

### 3.6 Nutrition practices

The maximum practice score was 15, while the minimum was 6, and the practice score was calculated based on 15 statements on nutrition practice. The mean ± SD and median of practice score were 10.77 ± 1.82 and 11 respectively. More than half of the athletes had a good level of practice (n = 150, 57.7%) while 42.3% (n = 110) had the practice score level poor (Figure 2).

Age had a significant relationship with the nutrition practice score of our participants. Good nutrition practices can be defined as practices that yield considerable changes in behavior and contribute to the improved nutritional status of the target population [26]. Examples of good nutrition practices include ‘eating fruits and vegetables’, ‘avoiding fast food’, ‘adding milk and dairy to the menu’. Participants, less than 15 years of age, had a better level of practices score than their seniors (p = 0.003). As with the age category, athletes with different BMI category shown to have significantly different practices (p = 0.004). Athletes categorize according to other variables like gender, educational level, department of training, training duration, the time before last training was completed and body fat percentage didn't show significantly different practice score (p > 0.05). Table 4 summarizes the detail.

### 3.7 Determinants of nutrition knowledge, attitude and practice

The determinants of nutrition knowledge, attitude, and practice score of athletes are shown in Table 4 and Table 5. The bivariate analysis showed that knowledge score was significantly different between athletes categorized according to age, gender, department of training and duration of training (Table 4). Whereas, none of the categories of athletes shown a significant difference in attitude score and practice score, whereas a significant difference in practice score was found between/among athletes when categorizing according to age and BMI.

**Table 5:** Unadjusted and Adjusted Odd Ratio of the association of different variables with knowledge, attitude and practice score.

Factors	Knowledge Score (Good vs Poor)		Attitude Score (Positive vs Negative)		Practice Score (Good vs Poor)	
	Unadjusted OR (*95%CI*)	Adjusted OR (*95%CI*)	Unadjusted OR (*95%CI*)	Adjusted OR (*95%CI*)	Unadjusted OR (*95%CI*)	Adjusted OR (*95%CI*)
**Gender** Female (Ref) Male	1 2.11 (0.94–4.75)	1 2.037 (*0.85–4.90*)	1 1.51 (0.68–3.36)	1 1.676 (*0.72–3.93*)	1 2.56 (1.12–5.83)∗	1 2.91 (*1.15 -7.37*) ∗
**Age** ≥15 vs < 15	1.78 (1.08–2.92)∗	1.119 (*0.59–2.11*)	1.12 (0.69–1.86)	0.996 (*0.53–1.88*)	0.59 (0.36–0.96)	0.609 (*0.32–1.18)*
**Weight (kg)**	1.02 (0.99–1.05)	1.59 (*1.14 -2.21*)∗	1.02 (0.99–1.05)	0.962 (*0.73–1.26*)	0.98 (0.95–1.0)	1.021 (*0.75–1.39*)
**Height (Inch)**	0.99 (0.93–1.05)	0.489 (*0.29-0.82*)∗	1.05 (0.99–1.12)	1.131 (*0.74–1.74*)	0.97 (0.92–1.03)	0.887 (*0.54–1.47*)
**BMI**	1.12 (1.0–1.24)∗	0.31 (*0.13 -0.77*)∗	1.02 (0.92–1.14)	1.129 (*0.53–2.41*)	0.92 (0.82–1.02)	0.926 (*0.39–2.23*)
**Body Fat (%)**	0.97 (0.92–1.03)	0.967 (*0.90–1.04*)	1.01 (0.95–1.06)	1.019 (*0.95–1.09)*	0.9 (0.85–0.95)∗	0.895 (*0.83 - 0.96*) ∗
**Dept. of Training** Cricket (Ref)	1	1	1	1	1	1
Football	0.79 (0.31–1.99)	0.63 (*0.23–1.73*)	0.76 (0.31–1.87)	0.778 (*0.30–2.01*)	2.36 (0.92–6.1)	1.694 (*0.60–4.77*)
Wushu	3.0 (0.59–15.29)	2.21 (*0.41–12.06*)	1.22 (0.35–4.29)	1.12 (*0.30–4.12*)	0.91 (0.27–3.05)	1.051 (*0.29–3.80*)
Hockey	0.75 (0.25–2.26)	0.508 (*0.15–1.67*)	1.02 (0.34–3.02)	0.939 (*0.30–2.92*)	2.12 (0.68–6.58)	2.045 (*0.60–6.96*)
Archery	0.46 (0.17–1.25)	0.571 (*0.2–1.63*)	0.99 (0.37–2.65)	1.058 (*0.38–2.93*)	0.979 (0.37–2.58)	1.003 (*0.35–2.88*)
Athletics	0.59 (0.24–1.46)	0.61 (*0.23–1.61*)	0.89 (0.36–2.19)	0.843 (*0.33–2.14*)	1.07 (0.44–2.59)	1.17 (*0.45–3.08*)
Basketball	0.39 (0.14–1.09)	0.18 (*0.05- 0.71*) ∗	0.87 (0.32–2.36)	0.617 (*0.18–2.17*)	1.16 (0.43–3.13)	1.171 (*0.32–4.24*)
Karate	0.5 (0.12–2.02)	0.471 (*0.11–2.02*)	0.45 (0.11–1.85)	0.457 (*0.11–1.92*)	0.23 (0.04–1.2)	0.278 (*0.05–1.59*)
Table Tennis	0.67 (0.23–1.96)	0.503 (*0.15–1.65*)	1.1 (0.38–3.24)	1.55 (*0.48–5.06*)	1.48 (0.51–4.3)	1.498 (*0.45–5.0*)
Taekwondo	0.5 (0.11–2.3)	0.501 (*0.11–2.39*)	0.1 (0.01–0.86)∗	0.095 (*0.01 -0.86*)∗	1.52 (0.32–7.17)	1.057 (*0.21–5.24*)
Volleyball	0.5 (0.17–1.48)	0.311 (*0.08–1.14*)	2.04 (0.62–6.67)	1.563 (*0.42–5.83*)	1.69 (0.56–5.07)	1.88 (*0.52–6.7*)

∗ = Significant, P value <0.05.

**Figure 3:**
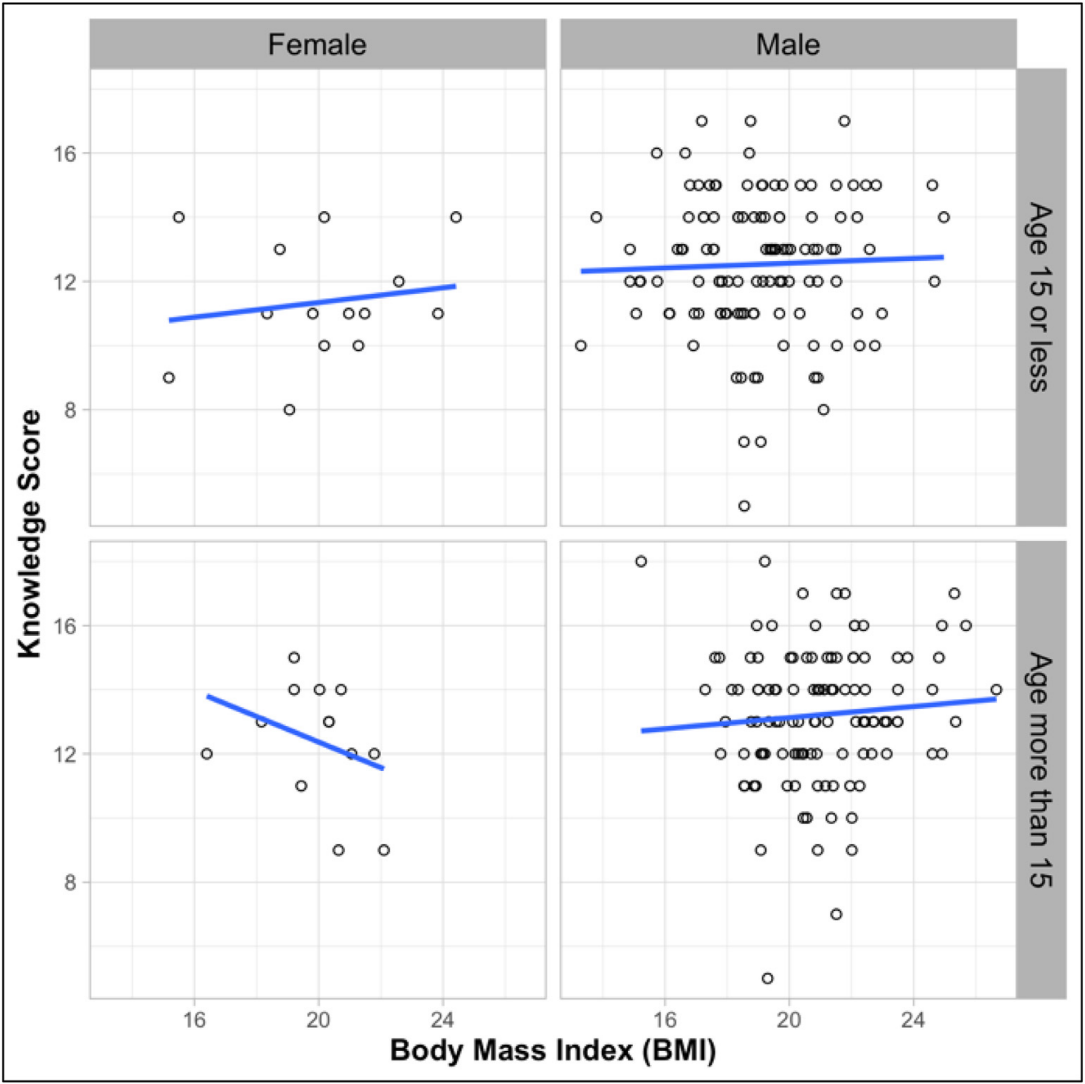
Association of Nutrition Knowledge Score and BMI of Athletes (According to Gender and Age category).

Multivariate logistic regression was shown to have a significant effect of age, weight, height, BMI, and department of training on knowledge score (Table 5, Figure 3). Athletes ≥15 years of age had greater chances of having a good nutrition knowledge score (UAOR 1.119; 95% CI 1.08, 2.92; p < 0.05) compared to athletes <15 years of age. However, after adjusting other variables, the effect of the age category became insignificant (p > 0.05). One kg increase in weight leads to a 1.59-fold increase in odd of having a good nutrition knowledge score (AOR 1.59; 95% CI 1.14, 2.207; p < 0.05). Unlike weight, every inch increase in height decreases the chance of having a good knowledge score (AOR 0.489; 95% CI 0.291, 0.822; p < 0.05). An increase in BMI also follows the same decreasing chance of having a good knowledge score (AOR 0.31; 95% CI 0.126, 0.766; p < 0.05). Athletes from the dept. of Basketball shown to have around one-fifth of the odd of having a good knowledge score (AOR 0.18; 95% CI 0.05, 0.071; p < 0.05) compared to athletes from the dept. of cricket.

**Figure 4:**
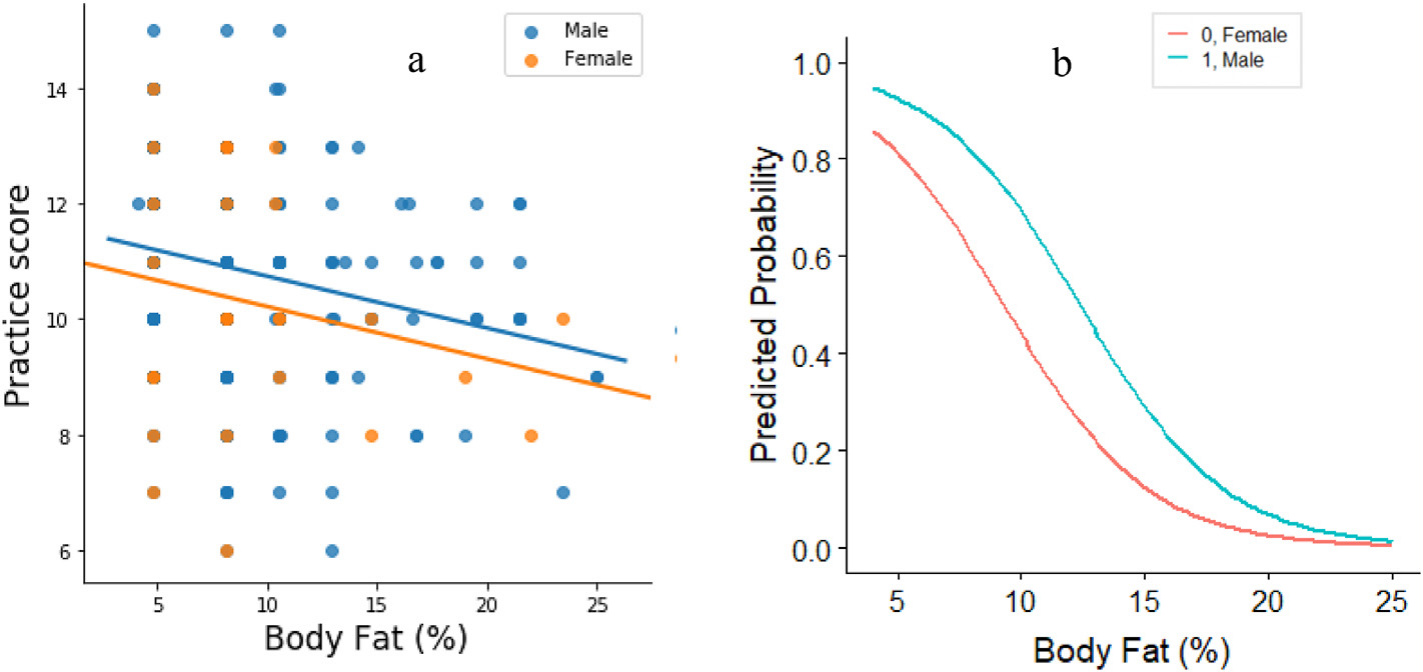
Correlation between practice score and percent body fat of athletes (Picture a) and Predicted Probability of having good practice score against percent body fat (Picture b). [Both are presented according to gender].

None of the variables shown to have a significant impact on the attitude score. Whereas, when practicing good nutrition habits, a male was shown to have around 3 times the odder of good practice score compared to the female counterpart (AOR 2.91; 95% CI 1.149, 7.373; p < 0.05). Athletes with more body fat were more likely to have less practice score (AOR 0.895; 95% CI 0.83, 0.96; p < 0.05) (Table 5 and Figure 4). As the body fat percentages of athletes decrease the predicted probability of having good practice score also increases and both male and female athletes follow this trend.

### 3.8 The relationship between knowledge and attitude score with practices score

**Figure 5:**
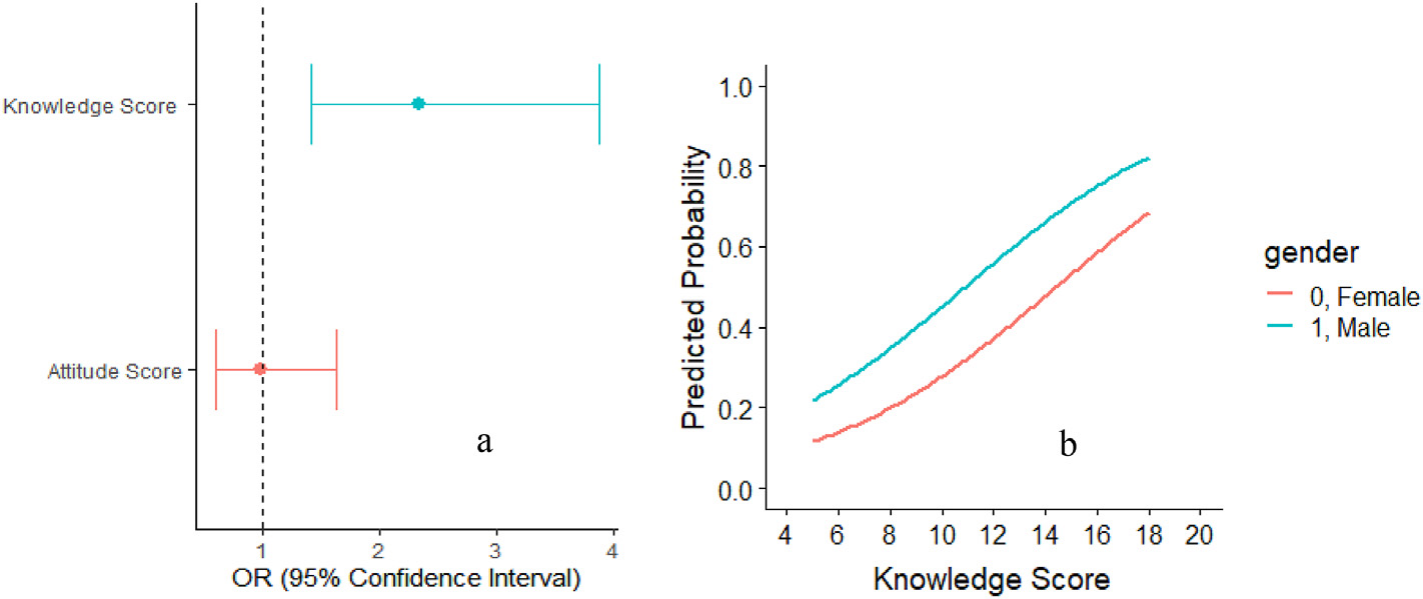
Adjusted Odd Ratio of the association of practice score with knowledge and attitude score (a) and Predicted Probability of having good practice score against knowledge score (Picture b). [Logistic model was developed adjusting height (inch), training duration and other variables used in the regression model].

The logistic regression model was developed to predict, how knowledge and attitude scores increase or decrease the chance of having good practice scores and shown in Figure 5. Athletes, those having good knowledge score were more than 2 times likely to have good practice score (AOR 2.335; 95% CI 1.405, 3.88; p = 0.001). The predicted probability of good practice score of athletes also increases as the knowledge score increases and this trend was true for both male and female athletes (Figure 4). Attitude score didn't show any positive or negative impact on practice score.

## 4 Discussion

Intake of proper food and adequate nutrition enhances athletic performance. The extent of exercise and intensity of training of an athlete also determines his/her nutritional requirements. Nutrition is an essential and integral part of a training program for proper fueling through optimal nutrition; most athletes nowadays understand this fact [27]. However, most of them lack proper knowledge and healthy dietary practices [6]. Therefore, this study investigated the current knowledge, attitude, and practices towards nutrition and their possible determinants and the relationship between each other among adolescent athletes of BKSP, Bangladesh.

From the result, we have found that the nutrition knowledge of our respondents was satisfactory as more than half of the participants had good nutritional knowledge. The mean knowledge score was similar to another study which had the same knowledge statements [28]. However, inadequate knowledge was observed in some other related studies [11]. Male participants had a higher level of nutrition knowledge score than females in a study [29]. The comparison was not statistically significant reported by another study [30]. Nevertheless, in our study, we have found a significant difference between genders in nutrition knowledge score. Males were more likely to have a good knowledge score (Table 4) than females. This insignificant difference in knowledge score between male and female athletes also reported by a few other studies [31, 32]. Athletes, more than 15 years of age had better knowledge scores compared to athletes less than 15 years of age (Table 4). However, the odds of having good nutrition knowledge were not significant among older athletes than the younger (Table 5). Nevertheless, the crude odds ratio shown that older athletes are more likely to have good nutrition knowledge. This adjustment effect could be explained as the effect of other variables included in the regression model. Rikrak Ch Marak et al. (2018) reported no differences in nutrition knowledge between older and younger athletes [31]. However, older athletes are more likely to have extra nutrition training or education compared to the younger ones. Heikkila, Maria, et al. (2018) also reported a similar effect of age on nutrition knowledge [33]. Athletes were more likely of having good nutrition knowledge with increasing their BMI (Table 5 and Figure 3). Nonetheless, when adjusting other variables, the relation reversed and athletes with lower BMI were more likely of having good nutrition knowledge. This may be due to the effect of other variables. Nutrition training had a significant association with participant's knowledge in some other studies [16, 30]. In this study, the mean knowledge score was higher among those participants who had previous exposure to nutrition training, but the difference was not significantly associated. (p > 0.05). The reason could be a vast number of participants who did not have any formal nutrition education before.

The primary source of information for nutrition were books, teachers, and coaches (Table 2). Very few of them sought nutrition advice from a nutritionist/dietician. The result was inconsistent with other studies, where most athletes preferred athletic trainers to dieticians to access nutrition-related information [34, 35]. Magazines, parents, teachers, and teammates were the commonly cited sources for nutrition revealed by another study [16] which is also similar to our results. However, such sources may not always be reliable. The quality of nutrition is vital than the number of available sources. This may also have been responsible for poor nutritional knowledge and routine dietary practices of athletes. To guide athletes better, the importance of coaches and trainers having appropriate nutrition information was emphasized [36]. Duration of the training period had a significant association with nutrition knowledge. Participants with a long duration of training scored better than their comparison group.

In the attitude section, the majority of them had a positive attitude towards nutrition (Figure 2). However, the mean score was relatively lower than a similar study having similar attitude statements [30]. We did not find any significant differences between age group, gender, educational level, BMI categories, and duration of the training, and previous exposure to nutrition training. This finding was following some other related studies [30]. Though another study had reported that females had higher attitude scores than their counterparts [37]. Nutrition knowledge had a significantly positive correlation with nutrition attitude reported by many studies [6, 16, 30, 37, 38, 39], and however, these findings was not in agreement with our finding. Around 3/5^th^ of our participants shown to have a positive attitude and the better attitude could be hypothesized due to good knowledge of athletes. Werblow et al. also reported a satisfactory attitude of athletes toward nutrition [40]. Several studies reported attitude as a strong predictor of performance and most of the athlete reported to believe a healthy diet as the important predictor of sports performance [41, 42, 43]. However, in the current study attitude was not significantly associated with nutrition knowledge and practice and no studied determinants were found to impact on attitude.

Practices to adhere to a healthy dietary lifestyle was also observed in most of our respondents, which were in accordance with another study having the same practice statements [26]. Nutrition knowledge was positively and significantly correlated with nutrition practices in this study. The result was in agreement with other previous studies [6, 11], but in contrast with another study [44]. Therefore, it is evident that proper education on nutrition not only increases knowledge but also have positive impacts on practices. Age had a significant relationship with nutrition knowledge and practices. Participants with a higher age group had a better level of knowledge, but participants with a lower age group in this study had a good level of practice. The reason could be lower age group participants tend to follow the diet schedule maintained at their residence and are more motivated. Nutrition knowledge, attitude, and practices improve with age except for the elderly observed by many other studies [45, 46]. This may be due to their education, extended presence in their respective training department, and life-related experiences. Gender-based differences in nutrition practices were observed and male tends to practice nutrition guidelines more (Table 5 and Figure 4a). However, our finding was not in agreement with other studies [12, 47]. It is often thought that females tend to be more conscious and knowledgeable regarding diet/nutrition-related issues [45], but this may not be the case in this study. This reverse output could be explained that the number of representative females was lowered compared to males (Table 1).

**Figure 6:**
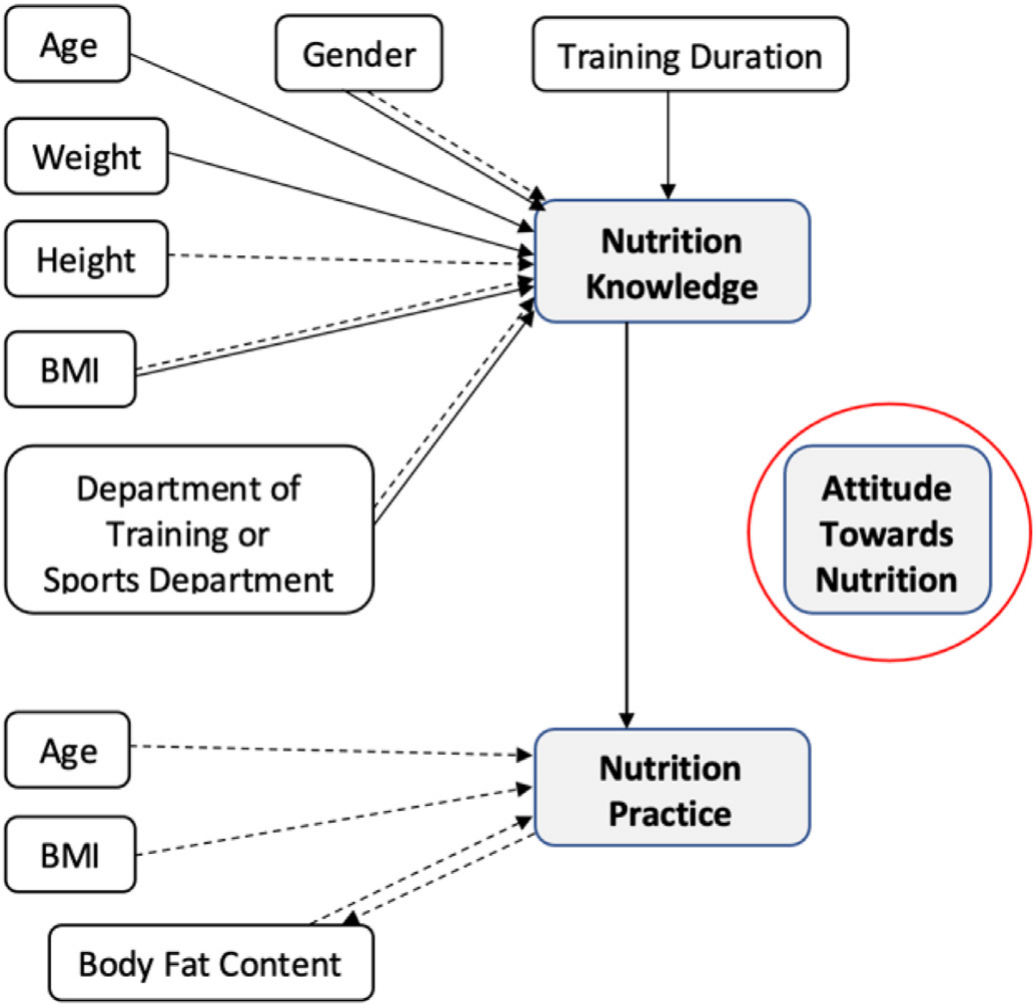
Determinants of Nutrition Knowledge, Attitude and Practice. (Flow diagram s developed from the output of bivariate analysis and multivariate logistic regression model) [Arrow means to have positive impact, while arrow with dash means negative impact and the red circle around the attitude means neither attitude have any determinants nor impact on knowledge and practice].

In the current study, athletes with higher body fat tend to practice nutrition guidelines less compared to those with lower body fat. This is a clear indication that athletes with lower body fat tend to practice more or otherwise athletes who practice more were able to keep their body fat minimum. However, nutrition knowledge and attitude were not related significantly with body fat percentages. This phenomenon could be explained that having good nutrition knowledge and attitude without practicing this in the real life is not enough to reduce body fat. Thus, it can be emphasized that having good nutrition knowledge and their implementation in the real life is important to get the best output in body fitness and athletic performance. This interrelationship can be better understood, when shedding light on the relation between knowledge score and practice score, as the odd and predicted probabilities of higher practice score was higher among athletes with higher knowledge score. This finding is in concur with some previous studies [41, 48, 49, 50]. Thus, the current findings indicate that's nutrition knowledge is positively associated with nutrition practice and modulating factors like duration of training could be a focal point of enchaining nutrition knowledge and practice behavior. Some factors like age, gender, and height have no opportunity to change, while training duration and department of training is significantly associated with nutrition knowledge, which in turns impact on nutrition practice and body fatness. These determinants (Figure 6) may play a key role in athletic performance. Thus, it is likely that the policymaker could emphasize nutrition training and training duration to enhance the performance of athletes.

### 4.1 Limitations and future research

The main limitation of this study was the small sample size. Moreover, dipropionate number of athletes from a different category of variables, like gender, religious group, BMI category, education level category, and so forth, pose potential problems in statistical validity. Moreover, every prediction equation has limitations as they may either over or underestimate percent body fat. There is no ideal prediction equation for body composition that is precise, accurate, affordable and practical. The predictive equation [25] used in the current study is not free from limitations. However, the strength of this equation is that the SEE values of validation remained <3%, the standard for body fat measurement as suggested by Lohman [51]. Another limitation was the use of the questionnaire; social desirability may diminish the validity of the responses. As our target populations were adolescent sports trainees, most of the participants had a healthy level of BMI. If a large number of adolescents from other schools who are underweight/overweight (not athletes) could be included, the result could have differed. Moreover, the dipropionate number of athletes from each of the strata, like the sports department, receiving training session, pose a problem in statistical analysis. Further, research, including analysis of specific food habits and the relationship between diet and diseases can help to determine appropriate nutritional education intervention in the long-term effect on health and athletic performance. Additionally, owing to a limited number of nutrition-related studies in Bangladesh, we suggest conducting more interventional studies to contribute to the promotion of nutrition education, aiming at improving an athlete's performance. Moreover, the findings of this study could be useful to generalize for athletes from a different area of Bangladesh, if further research was conducted ensuring a large sample consisting a proportionate number of athletes from different sports department and ensuring the inclusion of assessing nutrition knowledge of professional sportsperson or coaches.

## 5 Conclusion

In this research, the knowledge, attitude, and practices score observed in most of the respondents were satisfactory. Age, gender, sports department and duration of sports training received were significantly associated variables for nutrition knowledge, while only age and BMI were significantly associated variables for nutrition practices. For attitude towards nutrition, there were no such variables that had a significant association. Nutrition knowledge was significantly correlated with attitude and practices. We can conclude that promotion of nutrition knowledge through nutrition training subsequently can improve the attitude and practices. Therefore, the authority should pay attention to nutrition training. Regular education sessions (workshops, courses, seminars) for students by experts, including nutrition courses in the curriculum and coaches’ education on nutrition, are some of the key recommendations based on the results in this study. This emphasizes the need for the development and collaboration of sports nutritionists/dieticians in each sports department to bring positive changes in this area. The study faced few limitations as described in the discussion and disproportionate data is the important one. Thus, the study would get a better result with internal and external validity, if the more representative sample is included with a proportionated sample among different categorical variables.

## Declarations

### Author contribution statement

Bakhtiar, Masud-ur-Rahman, Shaikh Shahinur Rahman: Conceived and designed the experiments; Performed the experiments; Wrote the paper.

Kamruzzaman: Conceived and designed the experiments; Analyzed and interpreted the data; Wrote the paper.

Nargis Sultana: Performed the experiments.

### Funding statement

This research did not receive any specific grant from funding agencies in the public, commercial, or not-for-profit sectors.

### Data availability statement

Data will be made available on request.

### Declaration of interests statement

The authors declare no conflict of interest.

### Additional information

No additional information is available for this paper.

## Appendix A Supplementary data

Nutrition Questionnaire_HLY e06637_V2
