# Supplementary material for: Determinants of nutrition knowledge, attitude and practices of adolescent sports trainee: A cross-sectional study in Bangladesh
Source: Heliyon. 2021 Apr 6;7(4):e06637. doi: 10.1016/j.heliyon.2021.e06637 (PMC8056407; doi:10.1016/j.heliyon.2021.e06637)
Supplement: Supplementary file 1 — Nutrition Questionnaire_HLY e06637_V2 [file mmc1.pdf]

Nutrition knowledge, attitude and practices (KAP) of adolescent sports trainee in BKSP, Bangladesh

Questionnaire		
**Section A : Personal Questions**		
1. Age: ______________________	2. Gender ☐ Male ☐ Female	
3. Education : ☐School ☐College ☐University		
3. Weight: ______________	4. Height: ____________	5. BMI: _________________
6. How many nutrition class/seminar/course have you attended since you started your training?		
7. When was the last time you attended a nutrition class/seminar/course?		
8. From which sources have you obtained nutrition information?	☐ Books ☐ Magazines ☐ Newspapers<br>☐ Teachers ☐Coaches ☐ Athlete Trainers<br>☐ Doctors ☐ Dieticians ☐ Parents<br>☐ Friends ☐ Teammates ☐ Internet<br>☐Other(Please state__________________)	
9. Name of the Sports Department?		
10. Duration of Training period		

**Section B : Nutrition Knowledge** (For each of the following questions, tick the response that best answers the question)		
1. Protein is the main energy source for the muscle.	☐ True	☐ False
2. Fats have important roles in the body.	☐ True	☐ False
3. Iron-deficiency anemia results in a decrease in the amount of oxygen that can be carried in the blood.	☐ True	☐ False
4. Iron in meat is absorbed at the same rate as iron in a plant food.	☐ True	☐ False
5. The body can make vitamin D upon exposure to the sun.	☐ True	☐ False
6. Vitamin supplements are recommended for all physically active people.	☐ True	☐ False
7. During physical activity, feeling thirsty is enough to indicate the need for liquid.	☐ True	☐ False
8. Skipping meals is justifiable if you need to lose weight quickly	☐ True	☐ False
9. Foods like chocolate, biscuits and chips are the most appropriate foods to be consumed soon after training	☐ True	☐ False
10. Vitamins are good sources of energy	☐ True	☐ False
11. Alcohol consumption can affect absorption and utilization of nutrients	☐ True	☐ False
12. Saturated and unsaturated oils both have an equal effect on the health	☐ True	☐ False

13. Eating carbohydrates makes you fat	▢ True	▢ False
14. Dehydration decreases performance	▢ True	▢ False
15. The last meal before a competition should be eaten 3-4 hours before the competition	▢ True	▢ False
16. Males and females of the same age group spend equal amounts of calories during the same exercise	▢ True	▢ False
17. Bananas are good sources of potassium	▢ True	▢ False
18. Table salt is an essential part of a healthy diet	▢ True	▢ False
19. Milk and milk products are the best sources of calcium	▢ True	▢ False
20. Basic sugars like brown or granulated sugar, jam and honey are the most suitable energy sources for sportsmen.	▢ True	▢ False
21. Carbohydrates are stored in muscles in the form of glycogen	▢ True	▢ False
22. The last meal before a competition should be consumed 3-4 hours before the competition	▢ True	▢ False

Section C: Nutrition Attitude Questions (For each of the following questions, circle the number that best describes your answer, 1=Strongly agree, 2=Agree, 3=Undecided, 4=Disagree, 5=Strongly Disagree.

1. The relationship between good eating habits and good health should be stressed to the athlete.	1	2	3	4	5
2. Coaches need to have good attitudes toward nutrition because of their close contact and influence upon athletes.	1	2	3	4	5
3. The type of food an athlete eats affects his/her physical performance	1	2	3	4	5
4. What the athlete eats is only important if the athlete is trying to gain or lose weight.	1	2	3	4	5
5. Nutrition is more important during the competitive season than during the off-season for the athlete	1	2	3	4	5
6. Food advertisements are a very reliable source of nutritional information	1	2	3	4	5
7. It is the coach’s responsibility to stress good nutritional practices.	1	2	3	4	5
8. The athlete should schedule his/her activities so he/she has time to eat.	1	2	3	4	5
9. Learning about nutrition is not important for athletes because they eat so much food they always get the nutrients their bodies need.	1	2	3	4	5

10. Learning facts about nutrition is the best way to achieve favorable changes in food habits	1	2	3	4	5
11. Nutritional counseling would be important to the athlete who is trying to change his/her weight	1	2	3	4	5

**Section D: Nutrition Practice Questions**		
1. Do you use supplements like multivitamin as an athlete?	☐ Yes	☐ No
2. I consume lots of fruits and vegetables	☐ Yes	☐ No
3. I skip meals before a competition or an event	☐ Yes	☐ No
4. I eat just before an event	☐ Yes	☐ No
5. I eat just after an event	☐ Yes	☐ No
6. I consume sports drinks every day during practice or when I feel dehydrated	☐ Yes	☐ No
7. I eat adequate diet daily	☐ Yes	☐ No
8. I change my pattern of eating at the time of a competition	☐ Yes	☐ No
9. I always take my breakfast daily	☐ Yes	☐ No
10. I consume lots of water during and after training/competition	☐ Yes	☐ No
11. I always eat at least one hour before training/competition	☐ Yes	☐ No
12. I prefer snacks to special diet before training and competition	☐ Yes	☐ No
13. I eat at least 3times daily	☐ Yes	☐ No
14. I consume milk and milk products daily	☐ Yes	☐ No
15. I consume alcohol to enhance my performance	☐ Yes	☐ No
